# A network meta-analysis of risk factors of infection among close contacts of COVID-19

**DOI:** 10.1016/j.heliyon.2023.e20861

**Published:** 2023-10-10

**Authors:** Wei-wen Zhang, Chen-xi Li, Shu-jing Cao, Yu-yuan Wang, Ze-xi Lu, Jia-lin Sun, Ming -xia Jing

**Affiliations:** a Department of Preventive Medicine, School of Medicine, Shihezi University. Shihezi, 832003, PR China; b Key Laboratory for Prevention and Control of Emerging Infectious Diseases and Public Health Security, The Xinjiang Production and Construction Corps, PR China; c Oncological Department of Oral & Maxillofacial Surgery, The First Affiliated Hospital of Xinjiang Medical University, School／Hospital of Stomatology, Xinjiang Medical University, Stomatological Research Institute of Xinjiang Uygur Autonomous Region, Urumqi, 830054, PR China

**Keywords:** COVID-19, Close contact, Risk factors, Network meta-analysis

## Abstract

**Objective:**

We aimed to use network meta-analysis to compare the impact of infection risk factors of close contacts with COVID-19, identify the most influential factors and rank their subgroups. It can provide a theoretical basis for the rapid and accurate tracking and management of close contacts.

**Methods:**

We searched nine databases from December 1, 2019 to August 2, 2023, which only took Chinese and English studies into consideration. Odd ratios (ORs) were calculated from traditional meta-estimated secondary attack rates (SARs) for different risk factors, and risk ranking of these risk factors was calculated by the surface under the cumulative ranking curve (SUCRA).

**Results:**

25 studies with 152647 participants identified. Among all risk factors, the SUCRA of type of contact was 69.6 % and ranked first. Among six types of contact, compared with transportation contact, medical contact, social contact and other, daily contact increased risk of infection by 12.11 (OR: 12.11, 95 % confidence interval (CI): 6.51–22.55), 7.76 (OR: 7.76, 95 % CI: 4.09–14.73), 4.65 (OR: 4.65, 95 % CI: 2.66–8.51) and 8.23 OR: 8.23, 95 % CI: 4.23–16.01) times, respectively. Overall, SUCRA ranks from highest to lowest as daily contact (94.7 %), contact with pollution subjects (78.4 %), social contact (60.8 %), medical contact (31.8 %), other (27.9 %), transportation contact (6.4 %).

**Conclusion:**

The type of contact had the greatest impact on COVID-19 close contacts infection among the risk factors we included. Daily contact carried the greatest risk of infection among six types of contact, followed by contact with pollution subjects, social contact, other, medical contact and transportation contact. The results can provide scientific basis for rapid assess the risk of infection among close contacts based on fewer risk factors and pay attention to high-risk close contacts during management, thereby reducing tracking and management costs.

## 1 Introduction

The novel corona virus (Severe Acute Respiratory Syndrome Coronavirus 2, SARS-CoV-2) is in the midst of a global pandemic and it has produced a variety of mutant strains with different virulence and infectivity [1,2]. As of August 9, 2023, the number of confirmed cases of SARS-CoV-2 worldwide has reached 193209562, with 2958858 deaths [3]. The transmission of SARS-CoV-2 is primarily through droplet and airborne routes by respiratory activities [4,5], which has the characteristics of fast transmission, strong concealment of infectious source, and easy to cause large-scale transmission [6]. As people who have been in close contact with confirmed cases or with cases how are at an asymptomatic stage yet confirmed as SARS-CoV-2 carriers, have a higher risk of being infected by the virus [7,8]. Among various prevention and control measures such as masks use, self-testing and both conventional and rapid testing [7,[9], [10], [11]], close contacts tracking and management plays a very important role in controlling such infectious diseases. The key to determining the best plan to combat the epidemic lies in identifying and managing potential infected individuals in close contacts at a lower cost and in a shorter period of time, effectively blocking the spread of the epidemic and reducing its adverse effects [12].

During the outbreak of the epidemic, with the continuous detection of infected individuals and the large number of close contacts being tracked, enormous work pressure was placed on the transportation and isolation work of close contacts [13,14]. In previous cases of the epidemic, the positive rate of close contacts in centralized isolation was low, for example, a study in Guangdong, China showed that from February 9 to March 2, 2020, the close contacts number/cases number was 41.26, and the SAR of close contacts in centralized isolation was 1.13 % [15]; the SAR of close contact in Liaoning, China from April 24 to May 9, 2022 was 1.56 % [16]. This indicated that both in the early stages and with two years of prevention and control experience, there were situations where the range of close contact tracking was large and the effective isolation rate was low. It caused isolation resources wasted [15,17].

We can quickly assess the risk level of close contact infection based on fewer risk factors that are easily collected and have a significant impact on infection, and pay attention to high-risk close contacts in the management process, thereby reducing the time and cost of tracking and management, therefore, understanding the SARs of various infection risk factors and identifying important risk factors is crucial for rapid and accurate identification and management of close contacts [18,19]. Previous researchers have carried out a large number of studies on risk factors of infection among COVID-19 close contacts, however risk factors included in each study are not consistent and the reported secondary attack rates (SARs) varied largely. For example, the SARs for living together range from 3.83 % to 68.2 % [20,21], the SARs for social activities such as dining range from 1.02 % to 15.29 % [22,23], a difference of more than ten times. And there are also differences in the combined effects of some meta-analysis reports, for example, some studies believe that family members have a high risk of infection [12,24], while others believe that eating together has a high risk of infection [25]. Additionally, the understanding and evidence are lacking on the order in which different risk factors contribute to infection.

There is an appropriate approach to address this issue. Compared to traditional meta-analysis, network meta-analysis can produce indirect comparisons via a common comparator (i.e. evidence from studies with A vs. B and B vs. C, A and C can compared via B). Secondly, it can directly and indirectly compare multiple treatments and interventions, more accurately assessing the relative effects of different control groups to draw conclusions [26]. Thirdly, effect estimates from network meta-analysis can be linked to probabilistic modelling to obtain a relative ranking of interventions based on which is likely to be the most effective for the outcome of interest, which is likely to be the second best and so on [27]. Most epidemiological studies of infectious diseases are observational studies. When a research question in an observational study has multiple groups, the network meta-analysis principle can also be used to compare multiple groups [28].

Therefore, the purpose of this study is to comprehensively compare and rank the impact of various risk factors of infection in close contacts with COVID-19, determine the most influential factors of close contact infection, and rank its subgroups by using network meta-analysis. This study provides a provide scientific basis for the rapid and accurate identification and management of close contacts, effectively optimizes epidemic prevention resources, and provides reference for the prevention and control of similar emerging respiratory infectious diseases in the future.

## 2 Materials and methods

### 2.1 Study protocol

The network meta-analysis was performed using Preferred Reporting Items for Systematic Reviews and Meta‐Analyses (PRISMA). This study was registered with PROSPERO, number CRD 42022336859.

### 2.2 Definition

The classification of the type of contact was defined according to the Guideline of COVID-19 Control and Prevention-Close Contact Management Procedure (9th ed.) and was adjusted by included studies [29]. The type of contact was divided into six categories in this study: daily contact (working/living/studying in the same room), transportation contact, medical contact (contact in medical institutions, medical treatment and nursing), social contact (meal/entertainment/visiting/shopping contact, daily conversation, public place), contact with pollution subjects (exposure to environments and items contaminated by cases or asymptomatic infections) and other.

### 2.3 Search strategy

For this network meta-analysis, we searched PubMed, Web of Science, Embase, China National Knowledge Infrastructure (CNKI), WangFang Database, VIP, China Biology Medicine disc (CBM), medrixv and biorixv from December 1, 2019 to August 2, 2023, which only took Chinese and English studies into consideration. We developed and adapted a modified search algorithm for PubMed with combination of subject words and free words (in title and abstract) and then modified for other databases. The literature searching was completed by two reviewers. The search strategies for each database were in Supplementary Table 1.

### 2.4 Selection criteria

Studies meeting the following criteria were included: (1) Participants of study: close contacts of COVID-19 without medical workers; (2) Exposure: the article involves risk factors or prediction models of COVID-19 close contacts infected with SARS-CoV-2; (3) Outcome: SARS-CoV-2 infection; (4) Two or more risk factors of COVID-19 close contacts infection should be involved. The exclusion criteria were as follows: (1) Duplicate reports (in which case the study with the largest sample size or longest follow-up period was included); (2) Studies about epidemiological model for predicting the epidemic trend of COVID-19 and the case fatality rate; (3) Outcome indicators were not available or could be calculated by manual statistics; (4) Reviews, meeting abstracts, expert consensus and suggestions. The study selection was completed by two reviewers. Conflicts were resolved by a third reviewer.

### 2.5 Outcome measures

The primary outcome of network meta-analysis was the SARs of COVID-19 close contacts. SARs were defined as the probability that close contacts of index cases become confirmed cases of a disease [30]. The calculation formula is SARs = (the number of positive cases of the close contacts/the total number of the close contacts).

### 2.6 Data extraction

The data extracted from each report included title, the first author, publication time, study site, source of data, definition of close contacts, sample size, risk factors, number of contacts infected, number of contacts. Data abstraction was completed by independent pairs of reviewers. Conflicts were resolved by a third reviewer. Risk factors of COVID-19 close contacts infected in each study were classified into four categories based on the three keys to the epidemic of infectious diseases: source of infection, route of transmission and susceptible population. Finally, four categories of risk factors without overlap were included in our study: factors about index cases, factors about contact, factors about close contacts and factors about prevention and control measures. The risk factors were classified by two researchers independently. Disagreement was resolved by discussion to reach a third reviewer.

### 2.7 Quality evaluation

The Newcastle-Ottawa Scale (NOS) was used to evaluate the quality of included cohort and case‒control studies [31]. Each study was assessed on three domains (selection, comparability, exposure or outcome) included eight items. NOS used the semi-quantitative principle of star system to evaluate the quality of literature, with a total of 9 stars. The score was divided into three levels: low, medium and high quality, with corresponding scores of <5, 5–8, and 8–9. Two reviewers evaluated independently. Any discrepancy between two reviewers was resolved by discussion and adjudicated by a third reviewer when necessary.

### 2.8 Statistical analysis

We conducted network meta-analysis based on a random effects model. We used a network plot to show the relationship between the risk factors we included, calculated the relative rankings of risk factors and presented the surface under the cumulative ranking curve (SUCRA) percentages for both groups and subgroups. We selected and analyzed the type of contact based on the result of network plots and SUCRA. A network plot was used to compare the evidence among the type of contact, compared the type of contact by calculating the OR, and indicated which type of contact had the greatest impact on the infection of COVID-19 contacts according to SUCRA. To estimate inconsistency, we calculated the difference between indirect and direct estimates whenever indirect estimates could be constructed with a single common comparator. The Q test and I-squared (I^2^) metrics were used to evaluate the heterogeneity of the studies. Subgroup analysis was based on the study regions, investigation time, study type, literature quality, and sample size to analyze the sources of heterogeneity; For the sensitivity analysis, we remove included studies one by one to observe stability of results. Publication bias was examined by funnel plots and Egger's test. Network meta-analysis was performed by STATA version 14.0.

## 3 Results

### 3.1 Characteristics of included studies and literature quality

**Fig. 1:**
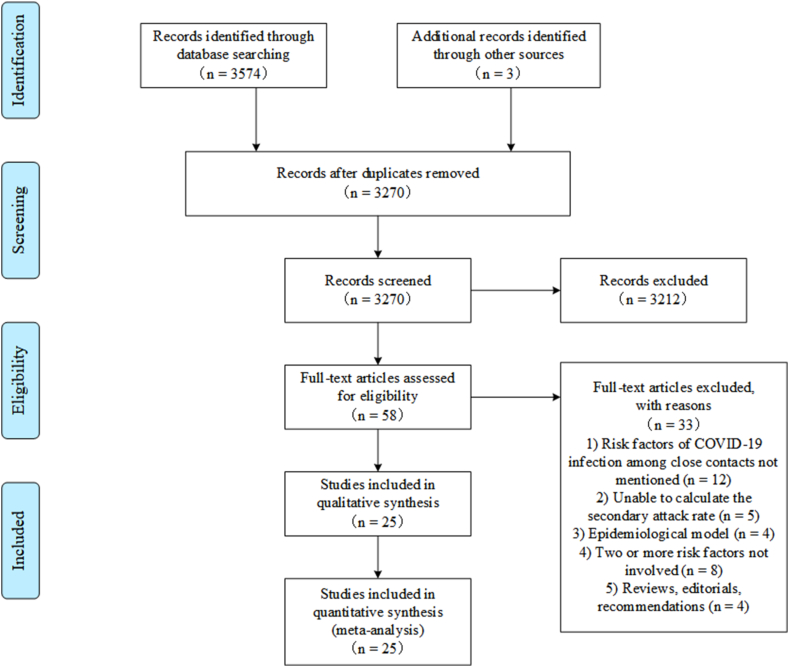
Flow diagram of the study selection process.

A total of 3577 studies were initially identified. After excluding duplicates, 3270 were reviewed by title and abstract, 58 studies progressed to full manuscript review, and 25 studies were included. The detailed process was illustrated in Fig. 1. 25studies involving 152647 close contacts were included in network meta-analysis. 5016 of them were infected. The studies were conducted in China (n = 19), Japan (n = 1), India (n = 1), Germany (n = 1), Iran (n = 1), Rwanda (n = 1), Singapore (n = 1). The studies we included involved a total of 18 risk factors, which were mainly divided into four categories: factors related to index cases (age of index cases, gender of index cases, occupation of index cases, symptomatic of index cases, N gene Ct value, severity of index cases), factors related to close contacts (age of close contacts, relationship with the index case, symptomatic of contacts), factors related to contact (type of contact，last contact time, distance to the patient, number of cases in contact, contact frequency, duration of contact, whether the cases outbreak at the time of exposure), factors related to prevention and control measures (surveillance method and mask of index case). The Supplementary File 2 presents the result of literature quality for the included studies. Of 5 case-control studies, 2 studies were judged to have 7 stars and 3 studies were judged to have 8 stars. Of 20 cohort studies, 13 scored 7 and 7 scored 8.10 studies were considered as high quality, and the rest were considered as medium quality.

### 3.2 Heterogeneity testing, sensitivity analysis, and subgroup analysis

The fixed effects model showed differences (I^2^ = 99.0 %, χ^2^ = 2390.13, P < 0.001), and a random effects model was used to merge the SARs (SAR = 0.071, 95%CI: 0.063–0.079, χ^2^ = 2390.13, P < 0.001, I^2^ = 99.0 %, Tau-squared = 0.0004). Sensitivity analysis showed robust results after excluding four studies. According to the study region, study data time, study type, literature quality, and sample size, no heterogeneity sources were found in subgroup analysis. The funnel plot and Egger method were used to test publication bias for all included studies, and the results showed the presence of publication bias (P < 0.001). The results of the trim and filling method showed that there is not much difference in the results before and after pruning, and the results are relatively stable. The inconsistency test showed the difference between direct and indirect evidence was not statistically significant (*p* > 0.05). There was no clear evidence suggesting inconsistency between direct and indirect network effects.

### 3.3 Outcomes of network meta-analysis

**Fig. 2:**
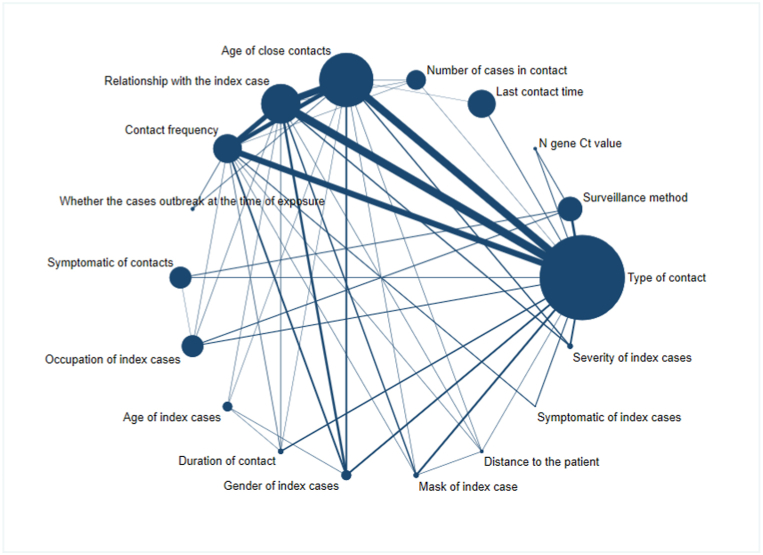
Network plot for risk factors of COVID-19 contacts infected.

Fig. 2 showed the comparative relationship between risk factors of SARS-CoV-2 infected. Each risk factor represented by a node. The size of nodes was proportional to the number of close contacts who infected SARS-CoV-2 related to that risk factor. The line between nodes indicated two risk factors were directly compared. The thickness of lines between risk factors related to the number of studies for that comparison. Fig. 2 illustrated most close contacts infected were related to type of contact in the included study population and there were a large number of studies on type of contact. In addition, there were lots of studies and sample sizes related to the age of close contact, the relationship with the indicated case, and the frequency of contact.

**Fig. 3:**
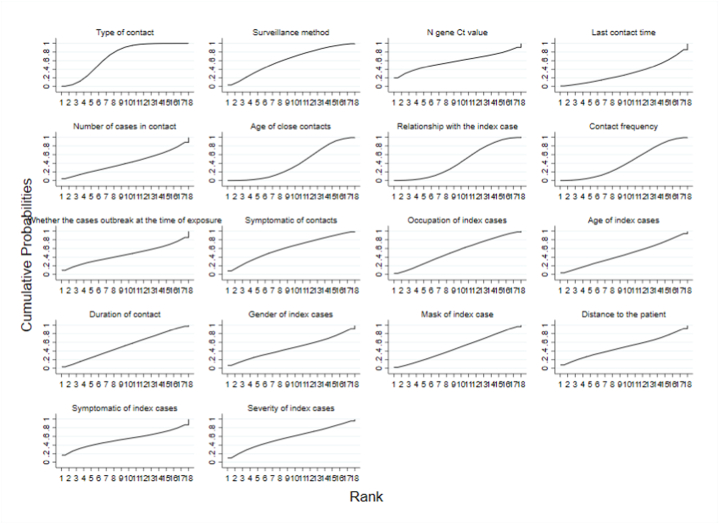
Cumulative ranking plots of risk factors of COVID-19 close contact(a total of 4 categories and 18 risk factors).

Cumulative ranking plots of each risk factor were shown in Fig. 3. The larger area under the curve, the greater impact of this factor on the results. The type of contact had the largest area under the curve (SUCRA = 69.6 %). Therefore, type of contact had the greatest impact on infection of COVID-19 close contacts. The top five also include surveillance method, symptomatic of contacts, N gene Ct value, severity of index cases.

**Fig. 4:**
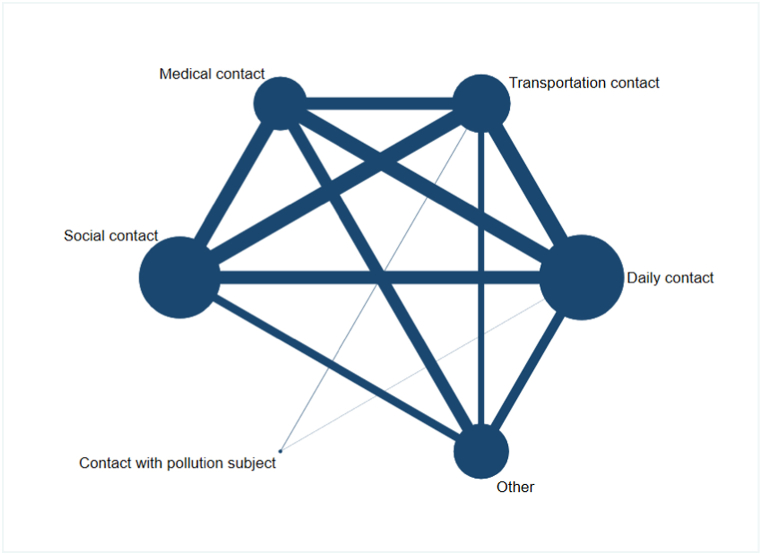
Network plot for the type of contact.

According to results of network plot and cumulative ranking plots, we further researched which type of contact had the greatest impact on infection of close contacts. The characteristics of included studies were summarized in Table 1. Among included studies, 20 studies included risk factors of infection among COVID-19 close contacts including type of contact, so we used these 20 studies for further exploration. Fig. 4 was the network plot for different types of contact. The maximum size node of daily contact showed that most close contacts infected were related to daily contact in the included study population and there were a large number of studies comparing daily contact, transportation contact, medical contact, and social contact.

**Table 1:** Meta-analysis of ORs in type of contact.

				Network meta-analysis		Traditional meta-analysis			
	Group	Number of studies	Sample size	SUCRA	Rank	OR (95 % CI)	I^2^(%)	Q test	*P*
All studies	Daily contact	20	34017	94.7	1	7.685(5.490–10.757)	93.8	306.03	<0.001
	Transportation contact	13	17857	6.4	6	0.176(0.105–0.297)	81.3	58.73	<0.001
	Medical contact	12	10830	31.8	4	0.395 (0.241–0.647)	82.5	62.74	<0.001
	Social contact	15	39903	60.8	3	0.627 (0.380–1.035)	95.2	290.30	<0.001
	Contact with pollution subject	1	1286	78.4	2	\	\	\	\
	Other	10	23656	27.9	5	0.250(0.141–0.442)	93.3	133.74	<0.001
Studies from China	Daily contact	12	59778	100.0	1	8.902(6.407–12.368)	92.6	231.29	<0.001
	Transportation contact	8	42180	7.2	5	0.176(0.105–0.297)	81.3	58.73	<0.001
	Medical contact	9	62847	41.2	3	0.395 (0.241–0.647)	82.5	62.74	0.007
	Social contact	9	47382	72.4	2	0.621(0.362–1.066)	95.5	290.12	<0.001
	Other	6	21884	29.2	4	0.216(0.122–0.385)	92.5	106.51	<0.001

*P* for Q test.

Table 2 detailed the complete matrix of result. The result showed pairwise comparison of the type of contact. Compared with transportation contact, medical contact, social contact and other, daily contact increased risk of infection by 12.11 (OR: 12.11, 95 % confidence interval (CI): 6.51–22.55), 7.76 (OR: 7.76, 95 % CI: 4.09–14.73), 4.65 (OR: 4.65, 95 % CI: 2.66–8.51) and 8.23 OR: 8.23, 95 % CI: 4.23–16.01) times, respectively. The risk of infection from transportation contact was 0.38 times higher than from social contact (OR: 0.38, 95 % CI: 0.19–0.76).

**Table 2:** The complete matrix of results.

Daily contact	0.08 (0.04,0.15)	0.13 (0.07,0.24)	0.21 (0.12,0.38)	0.54 (0.08,3.53)	0.12 (0.06,0.24)
**12.11 (6.51,22.55)**	**Transportation contact**	1.56 (0.74,3.28)	2.60 (1.31,5.17)	6.53 (0.99,42.87)	1.47 (0.66,3.29)
**7.76 (4.09,14.73)**	0.64 (0.30,1.35)	**Medical contact**	1.67 (0.84,3.31)	4.18 (0.59,29.55)	0.94 (0.43,2.05)
**4.65 (2.66,8.15)**	**0.38 (0.19,0.76)**	0.60 (0.30,1.19)	**Social contact**	2.51 (0.36,17.31)	0.57 (0.27,1.17)
1.86 (0.28,12.14)	0.15 (0.02,1.01)	0.24 (0.03,1.69)	0.40 (0.06,2.75)	**Contact with pollution subject**	0.23 (0.03,1.62)
**8.23 (4.23,16.01)**	0.68 (0.30,1.52)	1.06 (0.49,2.30)	1.77 (0.86,3.64)	4.44 (0.62,31.83)	**Other**

**Fig. 5:**
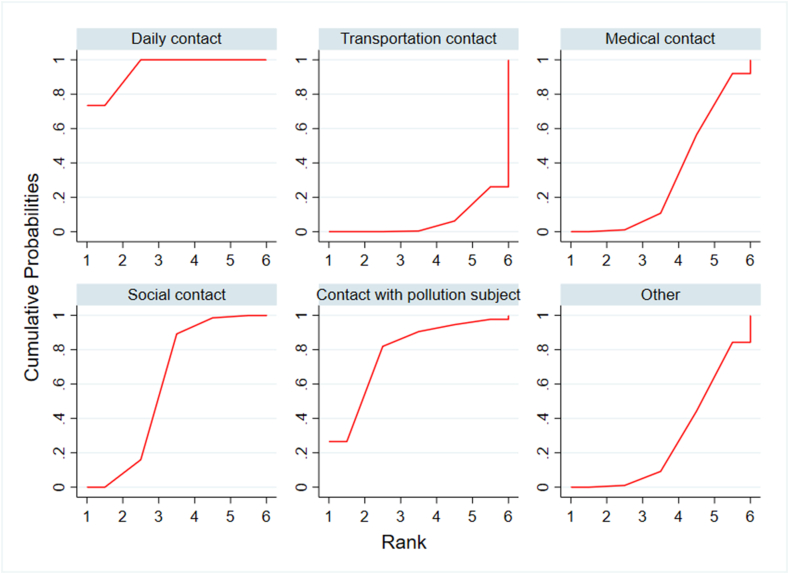
Cumulative ranking plots of six types of contact (daily contact, transportation contact, medical contact, social contact, contact with pollution subject and other).

Cumulative ranking plots for each type of contact were shown in Fig. 5. Among six types of contact, daily contact had the largest area under the curve (SUCRA = 94.2). Thus, daily contact has the greatest impact on the infection of COVID-19 close contacts, followed by contact with pollution subjects, social contact, other, medical contact and transportation contact.

We performed a subgroup analysis of studies in China, network comparisons and traditional meta-analysis for OR in the subgroup of China were shown in Table 1. The OR value of daily contact increased. Compared with other types of contact, daily contact had the highest OR (OR: 8.902, 95 % CI: 6.407–12.368). The OR value of social contact and other contact decreased and the OR of social contact was not statistically significant. Compared with the SUCRA of all studies, the relative ranks did not change.

**Fig. 6:**
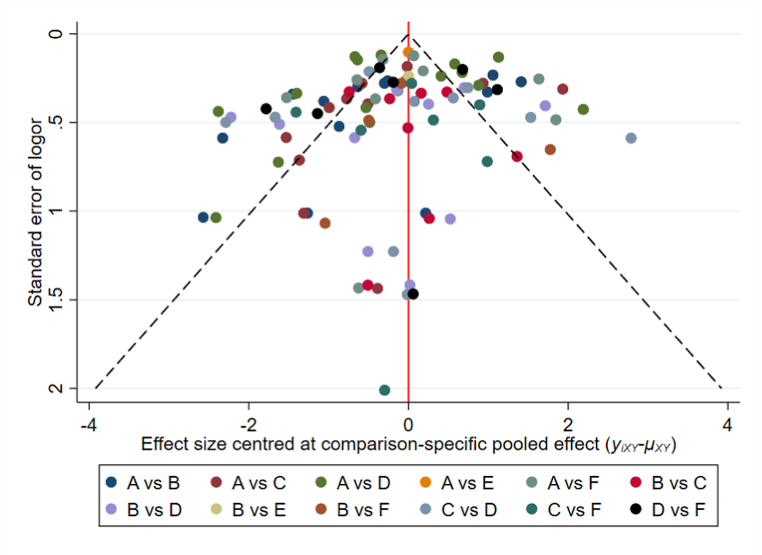
Funnel plots. NOTE: A: Daily contact, B: Transportation contact, C: Medical contact, D: Social contact, E: Contact with pollution subject, F: Other.

We performed heterogeneity test and sensitivity analysis on studies included type of contact, sensitivity analysis performed on each type of contact. The results of heterogeneity test were showed in Table 1. Sensitivity analysis demonstrated that the results were stable and reliable. Details were shown in the Supplementary Fig. 1. There was no apparent publication bias in the included studies (Fig. 6).

## 4 Discussion

### 4.1 Main finding

This study included 25 eligible observational involving 152647 participants of risk factors of infection among close contacts of COVID-19. The risk factors of infection were compared using network meta-analysis. Types of contact were further studied to reveal which types of contact cause the greatest risk of infection among close contacts according to the results. The infection risks of different types of contact were compared and the ORs were estimated. For all studies, types of contact had the greatest impact on infection of close contacts. And the OR of daily contact was significantly higher than transportation contact, medical contact and other. The SUCRA rankings from highest to lowest were daily contact, contact with pollution subject, social contact, medical contact, other and transportation contact, which implied among six types of contact, close contacts of COVID-19 were at greatest risk of infecting COVID-19 due to daily contact, followed by contact with pollution subject, social contact, medical contact, other and transportation contact.

The study we included involved a total of 4 categories and 18 risk factors. The factors about index cases mainly reflect the impact of source of infection. For example, after the elderly people are infected with SARS-CoV-2, they are prone to symptoms and progress to severe cases [32]. The virus can be excluded from the body through cough and other symptoms, infection may occur after close contact or inhalation of virus droplets [33]. Miao et al. found that close contacts of male index cases aged 41 to 60 had a significantly increased risk of infection [8]. The N gene Ct value response indicates the viral load of the case [34]. The factors about close contacts mainly reflect the influence of the susceptible population. The elderly population has poor immunity and is more likely to be infected with SARS-CoV-2 [35], Yu et al. found that infection rate increased with age [36]. The closer the relationship with the index cases, the more opportunities for close contact occur [37]. This agrees with the findings of Yang et al. which indicate that family members have the highest risk of infection [12]. The factors about contact reflects the impact of transmission routes. Type of contact represents the degree of contact with the index cases. Contact frequency and duration of contact represent the duration of exposure to the virus. The longer the time, the more the virus is inhaled. This agrees with the findings of Zhou et al. [38,39]. Distance is also an important factor, as droplets containing the virus have limited transmission distance after being exhaled by infected individuals. The closer the distance, the higher the virus concentration and the higher the risk of infection [40], Doung Ngern et al. also found that there was a negative correlation between social distance and infection risk [41]. Prevention and control measures reduce the spread of the virus, such as wearing masks can reduce the amount of virus discharged by infected individuals and also reduce the amount of virus inhaled by contacts [42,43]. SeyedAlinaghi et al. found that N 95 respirator had maximum efficacy, especially when used continuously [44].

Our study suggested that the OR estimates for each type of contact were more stable, with a smaller range of their 95 % CI after excluding studies from non-Chinese regions. The SUCRA ranking had no change. The ranking of type of contct had not changed. The main reason for this result is related to transmission characteristics of COVID-19. SARS-CoV-2 is transmitted primarily by the respiratory route (droplets and aerosols carrying pathogens) and direct contact with contaminated surfaces and fomites [45]. During living together, studying and working in the same room, long contact time, high frequency, close distance and no effective protective measures are prone to occur. It also provides opportunities for repeated, frequent and close contact with each other, which results in high recurrence, high infection, and high morbidity [23]. Thus close contacts of daily contact have the greatest risk of infection. Social contact includes having meals together, entertainment, meeting guests, and daily conversations. Although contact time is short, the density of people is large and the space is closed. The chance of transmission through air droplets, food contact and aerosols is greatly increased, and the risk of infection also increases. This is consistent with previous studies [37]. Among six types of contact, medical contact and transportation contact were associated with lower risk of infection. This probably because the prevention and control measures in medical institutions and transportation places are severe. In medical institutions, health care workers are required to use protective gowns, gloves, handwashing, eye protection, and multiple layers of personal protective equipment, which protect health care workers from infection to a certain extent. The cancelation of elective procedures and surgeries, the use of telemedicine, and the development of specialized COVID units in some health care facilities played an important role in limiting the transmission rates [[46], [47], [48]]. In the environment of stations, airports and transportation, people are asked to wear masks, temperature measurements, and check nucleic acid test reports, which reduce the possibility of infection. However, current studies highlighted that nucleic acid tests and antigen detection do not recognize patients but carriers of the virus, and there are several reasons for false-positives and false-negatives, that should be taken into account [[49], [50], [51]]. However, these two environments still need to be focused. Passengers can quickly reach distant places by means of transportation. If infection occurs, the virus can spread over a larger area in a shorter period of time. Medical institutions are more likely to contact with COVID-19 patients. Therefore, prevention and control measures in these places cannot be ignored.

There are some confounding factors in our study, which may lead to inaccurate result in the overall study analysis. First of all, different countries have different population densities, different criteria for judging COVID-19 close contacts and different national prevention and control policies. These regional factors will influence the infection risk of close contacts and may increase the heterogeneity of our study [52]. Secondly, the vaccination rate is an important confounding factor in our study, full-course vaccination, homologous booster vaccination and sequential vaccination will lead to better protection [53,54]. Therefore, the whole-course vaccination rate and booster vaccination rate also have an impact on the infection risk of close contacts. However, due to limitations of the original study, vaccination factors were not included. Finally, asymptomatic detection rate is also a confounding factor, compared with asymptomatic cases, symptomatic cases are at greater risk of transmitting the virus, their close contacts are at greater risk of infection [19,55,56]. Because a symptomatic infection can spread the virus into the air through symptoms such as coughing, it releases more virus during an episode of symptoms. If the asymptomatic detection rates and vaccination status of different type of contact are similar, confounding effects may be offset or attenuated.

### 4.2 Finding in different regions

The epidemic prevention and control policies in China were different from other countries, so subgroup analysis was carried out by region. After excluding studies from non-Chinese regions, the result of subgroup analysis suggested that OR for different types of contact changes. There may be several reasons. Most of study sites we included were from China, which was possibly because mainland China had more detailed and unified management and classification of close contacts than many other countries. After excluding studies from non-Chinese regions, estimates became significant and more accurate as the 95 % CIs narrowed, indicating stability and reliability of results increased. These differences may be caused by different standards for the definition and management of close contacts in China and other regions.

The types of contact classification varied and often simplified in some other countries and changed depending on the situation. China has conducted strict and detailed epidemiological investigations on close contacts, and divided all types of contact into six categories according to national guidelines. There have not been much changes in types of contact classification thus far. The World Health Organization guidelines for contact tracing classification were similar to Chinese guidelines, including household contacts, closed environment contacts, medical contacts, transportation contacts, and other well-defined contacts, but the latest criteria for close contacts do not have a clear type of contact classification [57]. The US Centers for Disease Control and Prevention provides risk assessment information and guidance for close contacts. As of 2021, the types of contact include school-work contact, transportation contact, community contact, and medical contact. The latest close contact definition does not provide a clear classification. It is simply divided into school contact and other environmental contact [58].

In addition, the Chinese government adopted strict social distancing measures in the early days of the epidemic, people were not allowed to go out unless necessary. Compared with one-off contact and certain social distance between social members in public places, living environment and long-term living conditions provide repeated, frequent, and close contact opportunities with each other, resulting in high recurrence, high infection, and high incidence of family members [[59], [60], [61]], which might also be a potential reason for significant results when calculating the OR between daily contact and other types of contact in the Chinese region.

We found that daily contact has the highest risk of SARS-CoV-2 infection, this contact is unavoidable. When people want to resume their previous social life, vaccines can help susceptible populations gain immunity, so vaccination may be a great way to reduce the risk of infection [[62], [63], [64], [65]]. For those who may have suboptimal response from COVID-19 vaccine, such as immunocompromised patients or patients with malignancy, alternatives such as monoclonal antibodies can be offered to provide extra protection [66].

This network meta-analysis has some limitations. First of all, due to language limitations, our study only included Chinese and English studies, and there was a selection bias in selection of included studies. Secondly, vaccination factors were not analyzed in the included studies. Although the protection of population by COVID-19 vaccine is not complete, it is also an important factor affecting close infection, which may be an important confounding factor. Homologous booster vaccination and sequential vaccination will make the COVID-19 vaccine more effective [53,54]. Therefore, we can further explore the impact of booster vaccination or sequential vaccination on controlling the COVID-19 epidemic. Thirdly, due to the limitations of included studies, we did not analyze other important confounding factors. Age, prevalent strains, criteria for COVID-19 close contact and intensity of control measures are all important confounding factors in this study, but this information was not described in included study. In terms of age, some only report population characteristics of close contacts as a whole, rather than population characteristics of each type of contact. Some reported only the demographic characteristics of subsequent cases, not all close contacts. Fourthly, considering the method limitations of network meta-analysis, at least two types of contact are required to be included in the study, so we excluded studies that reported only one type of contact and total SAR, which may lead to missing some information. Fifthly, our study ranked the impact of infection risk factors among close contacts of COVID-19, and then selected type of contact had the greatest impact on infection according to the results for further analysis. Due to the large number of variables, there are many other important risk factors waiting for us to analyze in the future. Finally, the heterogeneity analysis proved a significant heterogeneity of our study. Although we conducted subgroup analysis, we did not find the source of heterogeneity, this may be because different definitions of close contacts included in the study may lead to heterogeneity. The sample size of the studies included varies greatly, and there may be some heterogeneity when combining certain factors for analysis. The publication bias of SARs meta is inevitable, as it is equivalent to each study having a positive result during analysis, which may be one of the reasons for publication bias in all included studies.

## 5 Conclusion

The type of contact had the greatest impact on COVID-19 close contacts infection among risk factors we included. Daily contact carried the greatest risk of infection among six types of contact, followed by contact with pollution subjects, social contact, other, medical contact and transportation contact. The ORs of daily contact were significantly higher than other types of contact, supported by more accurate results using studies from China. Therefore, analyzing and ranking the infection risk factors of close contacts will tend to quickly make a decision according to one or several risk factors when tracing or managing, so as to take different management measures, reduce the waste of resources and the cost of tracking and management. In situations of resource scarcity, it is advisable to focus on close contacts with high infection risk levels, monitor their progression and take measures in time. It can also avoid the spread of epidemics due to loose management. This study is the foundation of our ongoing research. We are constructing an infection risk assessment model for close contacts of COVID-19, estimating the probability of close contacts based on factors that have a significant impact on infection, and dividing the risk level. This also has guiding significance for similar emerging respiratory infectious diseases in the future.

## Ethics approval

This article does not contain any studies with human participants or animals performed by any of the authors.

## Funding statement

This work was supported by Financial Science and Technology Project of the Corps (provincial level): The XPCC developed and demonstrated the application of a decision support platform for the precise prevention and control of major outbreaks.

## Data availability statement

Data included in article/supp. material/referenced in article.

## CRediT authorship contribution statement

**Wei-wen Zhang:** Writing – original draft, Methodology, Data curation. **Chen-xi Li:** Writing – review & editing, Methodology. **Shu-jing Cao:** Software, Data curation. **Yu-yuan Wang:** Software, Data curation. **Ze-xi Lu:** Software, Data curation. **Jia-lin Sun:** Writing – review & editing, Methodology, Conceptualization. **Ming -xia Jing:** Writing – review & editing, Conceptualization.

## Declaration of competing interest

The authors declare the following financial interests/personal relationships which may be considered as potential competing interests:Mingxia Jing reports financial support was provided by Financial Science and Technology Project of the Corps (provincial level).
