# Supplementary material for: A network meta-analysis of risk factors of infection among close contacts of COVID-19
Source: Heliyon. 2023 Oct 10;9(10):e20861. doi: 10.1016/j.heliyon.2023.e20861 (PMC10582502; doi:10.1016/j.heliyon.2023.e20861)
Supplement: Multimedia component 1 [file mmc1.docx]

**Supplementary Appendix**

**Supplementary Table 1. Strategies of search**

**Supplementary Table 2. Cohort study quality assessment results**

**Supplementary Table 3. Case-control study quality evaluation results**

**Supplementary Figure 1. Estimation of inconsistency**

**Supplementary Figure 2. SUCRA and cumulative probability plots**

**Supplementary Table 1. Strategies of search**

| Database | Specific strategy | Number of studies |
| --- | --- | --- |
| CNKIa | SU = (Novel coronavirus pneumonia + COVID-19) AND SU = (Close Contact) AND SU = (Risk Factor + Influencing Factor + Predictor + Predictive + Risk Prediction Model + Predictive Model + Risk Prediction + Risk Assessment) | 29 |
| Wanfang | Title or keywords: (Novel coronavirus pneumonia or COVID-19) and Title or keywords: (Close Contact) and Title or keywords:(Risk assessment or risk prediction or predictive model or predictive model or prediction or risk factor or predictor factor) | 15 |
| VIP | (M=Novel coronavirus pneumonia OR M=COVID-19) AND (M=Close Contact OR Contact) AND (M=Risk Assessment OR M=Risk Prediction OR M=Risk Prediction Model OR M=Risk Prediction Model OR M=Predictive OR M=Risk Factor OR M=Predictor Factor OR Influencing Factor) | 19 |
| CBMb | ( "Novel Coronavirus Pneumonia" [Title] OR " COVID-19" [Title]) AND ("Risk Assessment" [Title] OR "Risk Prediction" [Title] OR "Predictive Model" [Title] OR "Risk Prediction Model" [Title] OR "Risk Factor" [Title] OR "Influencing Factors" [Title]) AND ( "Close Contacts" [Title]) | 12 |
| PubMed | ((((((((((((((((COVID-19[MeSH]) OR (COVID19[Title])) OR (SARS-CoV-2 Infection[Title])) OR (2019 Novel Coronavirus Disease[Title])) OR (2019-nCoV Disease[Title])) OR (COVID-19 Virus Infection[Title])) OR (Coronavirus Disease 2019[Title])) OR (Severe Acute Respiratory Syndrome Coronavirus 2 Infection[Title])) OR (COVID-19 Virus Disease[Title])) OR (2019-nCoV Infection[Title])) OR (COVID-19 Pandemic[Title)) OR (SARS-CoV-2[Title])) OR (Coronavirus Disease 19[Title])) AND (close contact[Title] or contact) AND (((((((predictive model[Title]) OR (risk perdiction[Title])) OR (risk prediction model[Title])) OR (Predicting[Title])) OR (risk assessment[Title])) OR (risk factor[Title])) OR (Influencing factor[Title])) OR (Predictive factor[Title]))))) | 1304 |
| Web of Science | (((((((TS=(predictive model)) OR TS=(risk prediction)) OR TS=(risk prediction model)) OR TS=(predicting)) OR TS=(risk assessment)) OR TS=(Influencing factor)) OR TS=(predictive factor)) AND ((((((((((((TS=(COVID-19)) OR TS=(SARS-CoV-2Infection)) OR TS=(2019 Novel Coronavirus Disease)) OR TS=(2019-nCoV Disease)) OR TS=(COVID-19 Virus Infection)) OR TS=(Coronavirus Disease 2019)) OR TS=(Severe Acute Respiratory Syndrome Coronavirus 2 Infection)) OR TS=(COVID-19 Virus Disease)) OR TS=(2019-nCoV Infection)) OR TS=(COVID19)) OR TS=(SARS-CoV-2)) OR TS=(Coronavirus Disease 19)) AND (TS=(close contacts) OR TS=(contacts)) | 1762 |
| Embase | ('covid-19'/exp OR 'covid-19' OR 'sars-cov-2'/exp OR 'sars-cov-2' OR '2019 novel coronavirus disease'/exp OR '2019 novel coronavirus disease' OR 'covid-19 virus infection' OR 'severe acute respiratory syndrome coronavirus 2 infection' OR 'covid-19 pandemic') AND ('predictive mode' OR 'risk perdiction' OR 'risk prediction model'/exp OR 'risk prediction model' OR 'predicting' OR 'risk assessment'/exp OR 'risk assessment' OR 'risk factor'/exp OR 'risk factor' OR 'predictive factor'/exp OR 'predictive factor') | 340 |
| Biorxiv | TS=(predicting)) OR TS=(risk assessment)) OR TS=(Influencing factor)) OR TS=(predictive factor)) AND ((((((((((((TS=(COVID-19)) OR TS=(SARS-CoV-2Infection)) OR TS=(2019 Novel Coronavirus Disease)) OR TS=(2019-nCoV Disease)) OR TS=(COVID-19 Virus Infection)) OR TS=(Coronavirus Disease 2019)) OR TS=(Severe Acute Respiratory Syndrome Coronavirus 2 Infection)) OR TS=(COVID-19 Virus Disease)) OR TS=(2019-nCoV Infection)) OR TS=(COVID19)) OR TS=(SARS-CoV-2)) OR TS=(Coronavirus Disease 19)) AND (TS=(close contacts) OR TS=(contacts)) | 48 |
| Merxiv | TS=(predicting)) OR TS=(risk assessment)) OR TS=(Influencing factor)) OR TS=(predictive factor)) AND ((((((((((((TS=(COVID-19)) OR TS=(SARS-CoV-2Infection)) OR TS=(2019 Novel Coronavirus Disease)) OR TS=(2019-nCoV Disease)) OR TS=(COVID-19 Virus Infection)) OR TS=(Coronavirus Disease 2019)) OR TS=(Severe Acute Respiratory Syndrome Coronavirus 2 Infection)) OR TS=(COVID-19 Virus Disease)) OR TS=(2019-nCoV Infection)) OR TS=(COVID19)) OR TS=(SARS-CoV-2)) OR TS=(Coronavirus Disease 19)) AND (TS=(close contacts) OR TS=(contacts)) | 45 |
| aChina National Knowledge Infrastructure | | |
| b China Biology Medicine disc | | |

**Supplementary Table 2. Cohort study quality assessment results**

|  | | | | | | | | | |
| --- | --- | --- | --- | --- | --- | --- | --- | --- | --- |
| Study | Selection | | | | Comparability | Outcome | | | Scores |
|  | Representati-veness of the exposed cohort | Selection of the non exposed cohort | Ascertainment of exposure | Demonstration that outcome of interest was not present at start of study | Comparability of cohorts on the basis of the design or analysis | Assessment of outcome | Was follow-up long enough for outcomes to occur | Adequacy of follow up of cohorts |  |
| Lei Luo 2020 | ⭐ | ⭐ | ⭐ | ⭐ |  | ⭐ | ⭐ | ⭐ | 7 |
| Ng, O T 2021 | ⭐ | ⭐ | ⭐ | ⭐ |  | ⭐ | ⭐ | ⭐ | 7 |
| Binu Areekal 2021 | ⭐ | ⭐ | ⭐ | ⭐ | ⭐ | ⭐ | ⭐ | ⭐ | 8 |
| Peipei Hu 2021 | ⭐ | ⭐ | ⭐ | ⭐ |  | ⭐ | ⭐ | ⭐ | 7 |
| Yang Ge 2021 | ⭐ | ⭐ | ⭐ | ⭐ |  | ⭐ | ⭐ | ⭐ | 8 |

**Supplementary Table 3. Case-control study quality evaluation results**

|  | | | | | | | | | |
| --- | --- | --- | --- | --- | --- | --- | --- | --- | --- |
| Study | Selection | | | | Comparability | Exposure | | | Scores |
|  | Is the case definition adequate? | Represent-ativeness of the cases | Selection of Controls | Definition of Controls | Comparability of cases and controls on the basis of the design or analysis | Ascertainment of exposure | Same method of ascertainment for cases and controls | Non-Response rate |  |
| Shuangsheng Wu 2021 | ⭐ | ⭐ | ⭐ | ⭐ |  | ⭐ | ⭐ | ⭐ | 7 |
| Yunqi Miao 2021 | ⭐ | ⭐ | ⭐ | ⭐ |  | ⭐ | ⭐ |  | 6 |
| Shaoxia Song 2021 | ⭐ | ⭐ | ⭐ | ⭐ |  | ⭐ | ⭐ |  | 6 |
| Rui Zhang 2020 | ⭐ | ⭐ | ⭐ | ⭐ | ⭐ | ⭐ | ⭐ | ⭐ | 8 |
| Yu Ma 2020 | ⭐ | ⭐ | ⭐ | ⭐ | ⭐ | ⭐ | ⭐ | ⭐ | 8 |
| Wu Chen 2022 | ⭐ | ⭐ | ⭐ | ⭐ |  | ⭐ | ⭐ | ⭐ | 7 |
| Yang Li 2021 | ⭐ | ⭐ | ⭐ | ⭐ |  | ⭐ | ⭐ | ⭐ | 7 |
| Yongqiang Zhang 2021 | ⭐ | ⭐ | ⭐ | ⭐ |  | ⭐ | ⭐ | ⭐ | 7 |
| Ling Nie 2021 | ⭐ | ⭐ | ⭐ | ⭐ |  | ⭐ | ⭐ | ⭐ | 7 |
| Stich, M 2021 | ⭐ | ⭐ | ⭐ | ⭐ | ⭐ | ⭐ | ⭐ | ⭐ | 8 |
| Jashaninejad, R 2021 | ⭐ | ⭐ | ⭐ | ⭐ | ⭐ | ⭐ | ⭐ |  | 7 |
| Bi, Q 2020 | ⭐ | ⭐ | ⭐ | ⭐ | ⭐ | ⭐ | ⭐ | ⭐ | 8 |
| Semakula, M 2021 | ⭐ | ⭐ | ⭐ | ⭐ | ⭐ | ⭐ | ⭐ | ⭐ | 8 |
| Yoshikawa, H 2022 | ⭐ | ⭐ | ⭐ | ⭐ | ⭐ | ⭐ | ⭐ | ⭐ | 7 |
| Wu, Y 2020 | ⭐ | ⭐ | ⭐ | ⭐ | ⭐ | ⭐ | ⭐ | ⭐ | 8 |
| Ya Gao 2022 | ⭐ | ⭐ | ⭐ | ⭐ |  | ⭐ | ⭐ | ⭐ | 7 |
| Anran He 2022 | ⭐ | ⭐ | ⭐ | ⭐ |  | ⭐ | ⭐ | ⭐ | 7 |
| Haiqiang Jiang 2023 | ⭐ | ⭐ | ⭐ | ⭐ |  | ⭐ | ⭐ | ⭐ | 7 |
| Xiaoyun Yu 2023 | ⭐ | ⭐ | ⭐ | ⭐ |  | ⭐ | ⭐ | ⭐ | 7 |
| Fanglei Zhang 2022 | ⭐ | ⭐ | ⭐ | ⭐ |  | ⭐ | ⭐ | ⭐ | 7 |

**Supplementary Figure 1. Estimation of inconsistency**


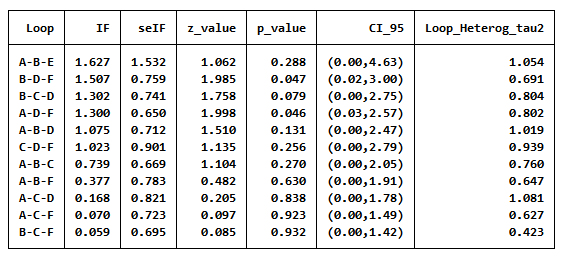


**Supplementary Figure 2. SUCRA and cumulative probability plots**

**2.1 Cumulative ranking plots of risk factors of COVID-19 close contact**


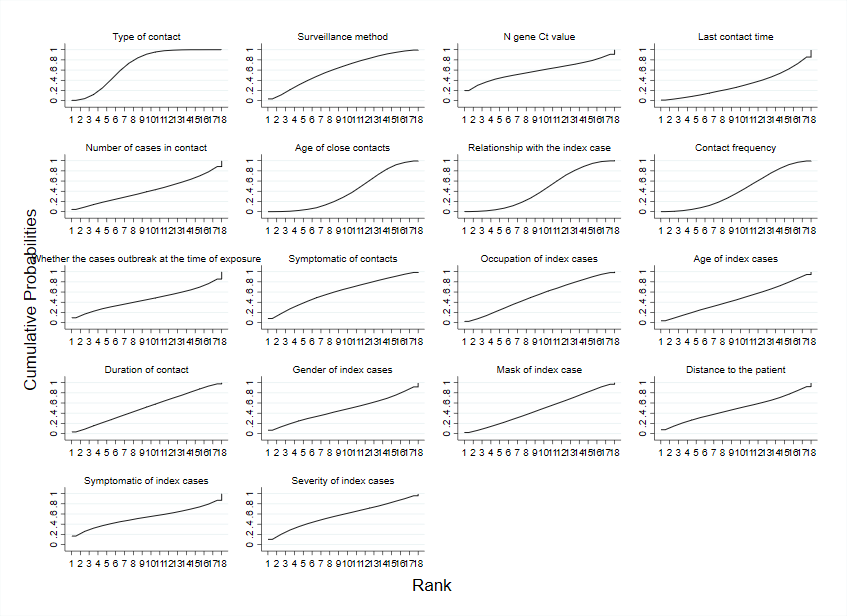

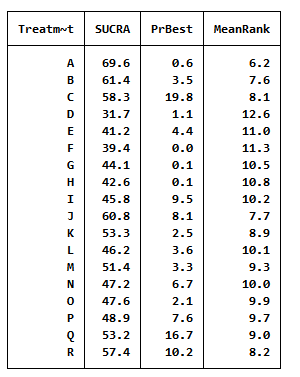


NOTE: A: Type of contact, B: Surveillance method, C: N gene Ct value, D: Last contact time, E: Number of cases in contact, F: Age of close contacts, G: Relationship with the index case, H: Contact frequency, I: Whether the cases outbreak at the time of exposure, J: Symptomatic of contacts, K: Occupation of index cases, L: Age of index cases, M: Duration of contact, N: Gender of index cases, O: Mask of index case, P: Distance to the patient, Q: Symptomatic of index cases, R:Severity of index cases

**2.2 Cumulative ranking plots of the type of contact**


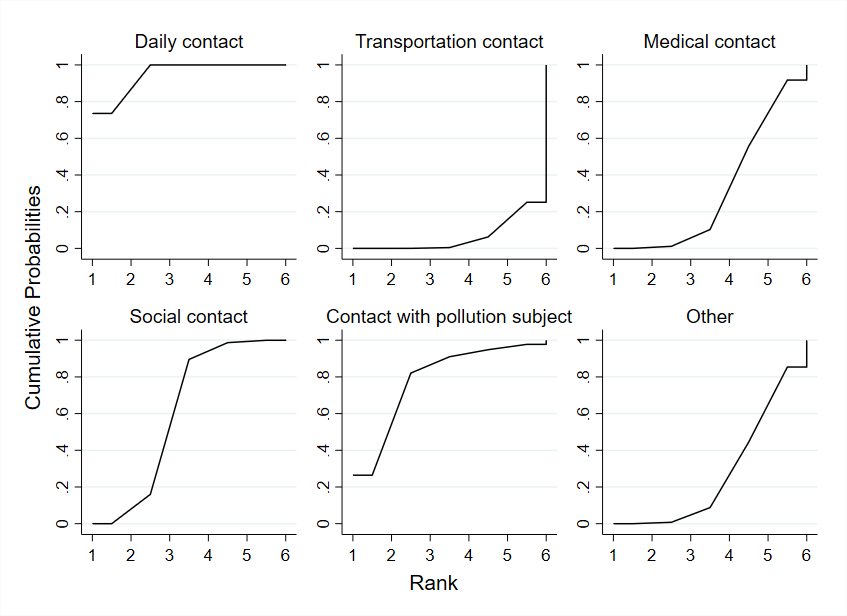

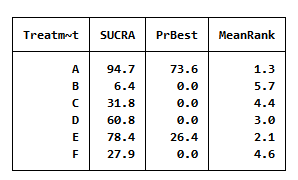


NOTE: A: Daily contact, B: Transportation contact, C: Medical contact, D: Social contact, E: Contact with pollution subject, F: Other
